# The Impact of Callous-Unemotional Traits and Externalizing Tendencies on Neural Responsivity to Reward and Punishment in Healthy Adolescents

**DOI:** 10.3389/fnins.2019.01319

**Published:** 2019-12-13

**Authors:** Yonglin Huang, Tingting Wu, Yu Gao, Yuyang Luo, Ziyan Wu, Shawn Fagan, Stephanie Leung, Xiaobo Li

**Affiliations:** ^1^Department of Psychology, Brooklyn College, The City University of New York, Brooklyn, NY, United States; ^2^Department of Psychology, Queens College, The City University of New York, Queens, NY, United States; ^3^The Graduate Center, The City University of New York, New York, NY, United States; ^4^Department of Biomedical Engineering, New Jersey Institute of Technology, Newark, NJ, United States; ^5^Department of Psychology, Pace University, New York, NY, United States

**Keywords:** callous-unemotional, externalizing, reward, punishment, adolescence

## Abstract

Both externalizing behavior and callous-unemotional (CU) traits in youth are precursors to later criminal offending in adulthood. It is posited that disruptions in reward and punishment processes may engender problematic behavior, such that CU traits and externalizing behavior may be linked to a dominant reward response style (e.g., heightened responsivity to rewards) and deficient punishment-processing. However, prior research has generated mixed findings and work examining both the sole and joint contribution of CU traits and externalizing problems related to functional brain alterations is lacking. In this pilot functional magnetic resonance imaging study, we measured externalizing behavior and CU traits in a community sample of adolescents (*n* = 29) and examined their impacts on brain activity associated with anticipation and receipt of reward and punishment using the Modified Monetary Incentive Delay task. We found that CU traits were associated with greater activation of the ventral striatum (VST) during reward anticipation. However, this effect became non-significant after controlling for externalizing behavior, indicating substantial overlap between the CU and externalizing measures in explaining VST activation when anticipating reward. In addition, externalizing behavior (but not CU) was significantly negatively associated with amygdala activation during punishment receipt, even after controlling for CU traits. The present findings extend previous evidence of hyper-responsivity to reward and hypo-responsivity to punishment in relation to psychopathic traits and antisocial behavior to non-clinical, non-incarcerated youths.

## Introduction

Early life presence of externalizing behavior and psychopathy are considered to be precursors to juvenile delinquency and later criminal offending. In adults, psychopathic traits are a constellation of personality characteristics comprised of callousness, lack of empathy, superficiality, and impulsivity (Hare, 2003). Research has extended the concept of psychopathy to youth by identifying the core traits (lack of empathy and/or guilt, shallow and limited affect), which are referred to as callous-unemotional (CU) traits (Frick, 1995; Barry et al., 2000; Frick et al., 2014). Externalizing behavior, including aggressive and rule-breaking behaviors, are behaviors that violate societal norms and infringe on others’ rights (Liu, 2004; Calkins and Keane, 2009). The terms “externalizing behavior” and “antisocial behavior” have been used interchangeably by some researchers, although others argue that “externalizing behavior” should be reserved to characterize destructive behaviors exhibited by youth that are less severe than antisocial behaviors such as negative, hostile, and defiant acts (Shaw and Winslow, 1997; Liu, 2004). Externalizing behavior and CU traits are highly correlated (e.g., Charles et al., 2012; Pihet et al., 2015). Furthermore, the presence of CU traits in youth is associated with more severe externalizing behavior (e.g., conduct problems, delinquency, aggression) (see Frick and Dickens, 2006 for a review), and is especially useful for predicting a subgroup of individuals with antisocial behavior that are more serious and chronic in nature (Frick and White, 2008). In fact, the Diagnostic and Statistical Manual of Mental Disorders (DSM-5; American Psychiatric Association [APA], 2013) includes a CU specifier as a feature of conduct disorder [a disorder in youth characterized by externalizing behavior that tends to precede antisocial personality disorder (ASPD)]. ASPD is a personality disorder marked by persistent disregard and/or violation of other’s rights, and is often accompanied by criminal and aggressive behavior (American Psychiatric Association [APA], 2013). While only 5% of youth exhibit a pervasive and egregious pattern of both CU traits and externalizing/antisocial behavior, by adulthood this 5% accounts for a staggering 50% of crime in the United States alone (Loeber et al., 2000; Hinshaw and Lee, 2003). Therefore, it is essential to understand the etiology of CU traits and externalizing behavior in youths to help combat the future development of maladaptive behaviors.

One potential etiological pathway that engenders externalizing behavior is hyperactivity to rewards and/or hypoactivity to punishment (Frick and Marsee, 2006; Pardini, 2006; Byrd et al., 2014). Specifically, reward oversensitivity in externalizing youth can result in persistent reward-seeking behavior (Quay, 1993), and antisocial youth may rely more heavily on acting on appetitive drives than evading punishment (O’Brien and Frick, 1996). Meanwhile, insensitivity to punishment has also been implicated in psychopathy and antisocial behavior (Dadds and Salmon, 2003; Hawes and Dadds, 2005). Deficits in punishment processing may result in failure to adopt appropriate behavior via passive avoidance learning (Newman and Kosson, 1986; von Borries et al., 2010), whereby externalizing/antisocial behavior may be the behavioral manifestation of punishment insensitivity (Lykken, 1995). Impaired punishment processing (i.e., hypo-responsivity) may lead to lower levels of anxiety and fear anticipation, thereby giving rise to psychopathic and antisocial tendencies. One conceptualization has postulated that psychopathy and externalizing/antisocial behavior may arise from deficits in the modulation of both reward and punishment systems (Patterson and Newman, 1993; Wallace and Newman, 2008; Byrd et al., 2014).

Processing of reward can be distinguished by two phases: anticipation of a reward and its delivery (Knutson et al., 2001b). Evidence from functional magnetic resonance imaging (fMRI) studies on healthy populations has suggested that similar regions are activated during reward anticipation and receipt (e.g., insula, dorsal and ventral striatum) (Silverman et al., 2015), with the ventral striatum (VST) being critically implicated during the processing of reward (Knutson et al., 2001b; Oldham et al., 2018). Similarly, an anticipatory phase and the delivery of the punishment are also included in the processing of punishment (Delgado et al., 2009): anticipation elicits activation of the VST, amygdala, thalamus, and insula (Oldham et al., 2018), while punishment receipt activates the anterior cingulate cortex (ACC), insula, thalamus, and orbitofrontal cortex (OFC) (Wrase et al., 2007). One recent study indicates that overlapping neural substrates, including VST, thalamus, and amygdala are implicated in both reward and punishment anticipation (Oldham et al., 2018). Taken together, anticipation and receipts of rewards and punishments may implicate brain regions that are largely overlapping, but also distinctive.

Altered function in many of these regions has been linked to reward and punishment processing deficits seen in antisocial and psychopathic adults (Hyde et al., 2013). Meanwhile, hyposensitivity to punishment and/or hypersensitivity to reward have largely been implicated in the development of externalizing behavior and CU traits in youths (Matthys et al., 2004; Rubia et al., 2009; Bjork et al., 2010), although abnormalities in various regions have been reported. For example, in one study, adolescent males with early onset conduct disorder showed decreased activation of the OFC during reward receipt (Rubia et al., 2009). In another study, adolescents diagnosed with either oppositional defiant disorder (ODD), attention deficit hyperactivity disorder (ADHD), or conduct disorder (CD) exhibited greater activation in nucleus accumbens (NAcc; considered to be a primary subregion of the VST) and the ACC in response to reward receipt (Bjork et al., 2010) when compared to healthy controls. In contrast, an investigation on 16- to 19- year-old adolescents with disruptive behavioral disorders (DBD) had compared DBD persisters (who showed early onset and persisted into late adolescence/adulthood), DBD desisters (who showed late onset and eventually ceased disruptive behaviors) and healthy controls (Cohn et al., 2015). They found that the DBD persisters showed blunted activation in the VST, but increased activation in the amygdala, to the receipt of the monetary gain, as compared to the other two groups. In addition, CU traits were associated with reduced amygdala activation in response to monetary receipt (Cohn et al., 2015). Finally, in boys aged 8- to 11- years with clinically significant conduct problems, no significant association was found between CU traits and neural response to reward receipt in any of the above regions (Byrd et al., 2018). Overall, most of the research on youth samples support the proposition that CU traits and externalizing tendencies are associated with hyper-responsivity to reward, although more work is warranted to address prior mixed findings.

In regards to punishment, several studies have found impaired aversive conditioning in psychopathic, CU, and externalizing populations, which is consistent with the punishment hypo-responsivity theory. Yet, studies have also yielded conflicting results and few have looked at both CU traits and externalizing behavior together. Youth with externalizing behavior (Gao et al., 2009, 2010b), conduct disorder (Fairchild et al., 2008), and those with CU traits from the community (Fung et al., 2005) have lower skin conductance responses (SCRs), an index of autonomic arousal, to cues that signaled punishment, indicating their lack of fear for impending punishments or risks. Studies that examined responses to the receipt of punishment have yielded comparable results to those of impending punishment. Youth with DBD showed reduced eye-blink startle responses (van Goozen et al., 2004) and reduced SCRs to uncued aversive tones (Herpertz et al., 2003) than normal controls. In youth with DBD and CU traits, reduced amygdala activation in response to punishment has been reported (Finger et al., 2011), but failed to replicate in another study (Byrd et al., 2018). However, Byrd et al. (2018) did not find any association between conduct problems and reductions in amygdala activation following punishment. In contrast, Cohn et al. (2015) found that DBD persisters had increased amygdala activation to punishment feedback.

Although not specifically related to reward and punishment, there is burgeoning evidence for an interactive role of CU traits in relation to brain deficits in externalizing adolescents. CU traits in youth with disruptive behavior have been associated with atypical brain functioning, particularly reduced amygdala response to socio-affective cues (e.g., fearful expressions) (Marsh et al., 2008; Jones et al., 2009; Viding et al., 2012; White et al., 2012), which in turn may lead to increases in proactive, goal-directed, aggressive behavior observed in youths with CU traits (Lozier et al., 2014). Alternatively, youths with behavioral problems and unspecified CU traits exhibit elevated activation in the amygdala, insula, and striatum in response to socio-affective stimuli (Herpertz et al., 2008; Passamonti et al., 2010). Research has shown that while CU traits are positively correlated with externalizing behavioral problems, these variables are, respectively, negatively and positively correlated with amygdala responses to socio-affective stimuli (Lozier et al., 2014). Moreover, externalizing boys with high CU traits exhibited amygdala hypo-reactivity to fearful faces, whereas externalizing boys with low CU traits were hyper-reactive (Viding et al., 2012). A more recent study reported similar results: youth with high CU traits showed amygdala hypo-reactivity when making judgments about causing fear in others and CU traits moderated the relationship between externalizing behavior and both the functional connectivity and activity of the amygdala (Cardinale et al., 2018). CU traits were also found to account for structural abnormalities in the amygdala of children with externalizing problems (Cardinale et al., 2019). While preliminary, these results suggest that CU traits and externalizing symptoms may play an interactive role in predicting brain deficits in youths.

In this pilot study, we aimed to extend the prior findings to non-clinical, non-incarcerated adolescents. Not only are non-clinical, non-incarcerated youth underexplored, but further examination of this group is important in identifying potential risk factors for the development of externalizing behavior and CU traits. Externalizing behavior (via the Child Behavior Checklist, CBCL; Achenbach, 1991) and CU traits (via the Inventory of Callous Unemotional Traits, ICU; Frick, 2004) were assessed in a group of adolescents from the community. They completed a modified Monetary Incentive Delay task (MID; Knutson et al., 2001a), in which participants were shown three types of geometric cues, each associated with either potential gain, loss, or no gain or loss (neutral). Their brain activation during anticipation and receipt of monetary gain and loss were acquired. Studies on healthy adolescents and adults have shown involvement of the NAcc, caudate, putamen, thalamus, and insula during the reward and loss anticipation phases of the MID (Cho et al., 2013). We expected to see hyper-responsivity to reward and hypo-responsivity to punishment in relation to externalizing and/or CU tendencies. Given that the presence of CU traits may designate a subgroup of youth with more severe externalizing tendencies (Frick and White, 2008), our second aim was to test if CU traits would interact with externalizing behavior in predicting brain activation in response to reward and punishment. It was hypothesized that adolescents with higher levels of both would show the most aberrant neural responses (e.g., hyper-responsivity to reward and hypo-responsivity to punishment).

In addition, given that prior studies have reported high correlations between externalizing behavior and CU traits (Charles et al., 2012; Pihet et al., 2015) and that they share similar etiological profiles (Frick and Dickens, 2006), we investigated the unique contribution of CU traits and externalizing behavior to reward/punishment processing in this pilot study. We predicted that there would be a great amount of overlap between externalizing behavior and CU traits in explaining brain activation. Finally, since prior research has implicated low IQ and high social adversity in antisocial behavior (e.g., Moffitt et al., 1981; Fagan et al., 2017), we included measures of IQ and social adversity as covariates. Sex and pubertal status were also included as covariates given that sex differences in the structural and functional abnormality have been found in antisocial populations (e.g., Raine et al., 2011; Visser et al., 2013) and an increasing number of studies have illustrated the effects of puberty on brain structure (Urošević et al., 2014; Herting and Sowell, 2017). To the authors’ knowledge, this is the first study to investigate an interaction effect of CU traits and externalizing behavior in relation to reward and punishment processing deficits in youths.

## Materials and Methods

### Participants

Data were collected as part of the Healthy Childhood Study (HCS), an ongoing longitudinal study that follows healthy children through development. Participants and their families were originally recruited from the metropolitan Brooklyn, New York community when children were 7- to 10- years old. The original cohort consisted of 340 participants [48.2% male, mean age = 9.06, standard deviation (SD) = 0.60] and their main caregivers. Youth participants with any history of drug use, psychiatric disorders, intellectual disabilities, or developmental disorders were excluded from recruitment. More details of the full cohort can be found in Gao and Zhang (2016).

From the original cohort, 32 adolescents were randomly selected and invited to participate in the current fMRI study when they were 11- to 14-year-old. Three were excluded due to excessive head motion (>8 mm in translation or >5° in rotation) during the task. The remaining 29 adolescents were comprised of 15 girls and 14 boys (mean age = 12.3, *SD* = 0.8). Twenty-three of them were right-handed. The ethnic breakdown was as follows: 58.6% Black, 24.1% Hispanic, 13.8% Caucasian, and 3.4% mixed-race/other. Caregiver participants consisted of biological mothers (86.2%) and biological fathers (13.8%).

Participants and their main caregivers were invited to the Translational and Molecular Imaging Institute of Icahn School of Medicine at Mount Sinai (ISMMS) in New York for the interview assessments, the mock scan, and the actual scan, which lasted approximately 2 h in total. Participating families were financially compensated $150 for their participation. All procedures were approved by the Institutional Review Boards of the City University of New York and the ISMMS. Written informed parental consent and youth assent were obtained from each family before participation. After consenting, caregivers filled out the CBCL and ICU. Youths filled out the ICU and Self-Rating Scale for Pubertal Development (see below) after the brain scan.

### Measures

#### Externalizing Problems

**FIGURE 1:**
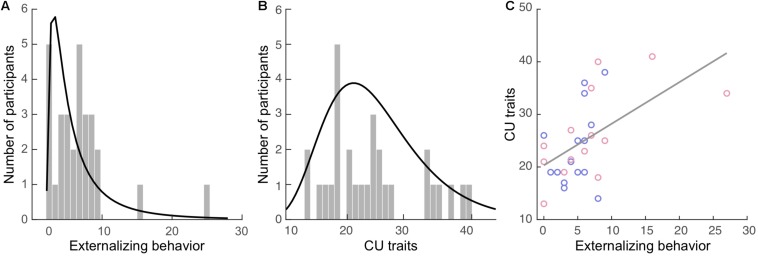
Externalizing behavior and callous-unemotional (CU) traits in the current sample. Histograms of the **(A)** externalizing behavior and **(B)** CU traits. The curves in black represent the fitted distributions. **(C)** Positive correlation between these two measures. Blue dots: boys. Red dots: girls.

Externalizing behavior was measured via caregiver’s report using the Child Behavior Checklist (CBCL; Achenbach, 1991). The CBCL contains 112 items concerning a child’s behavior, including internalizing (77 items) and externalizing behavior (35 items), within the past 12 months. The externalizing subscale is comprised of the aggression (e.g., “Cruelty, bullying, or meanness to others”), and delinquency (e.g., “Breaks rules at home, school, or elsewhere”) subscales. Items are rated on a 3-point scale ranging from 0 (not true) to 2 (very true or often true). The total externalizing score was computed as the sum of all relevant items for each participant. Internal consistency of the externalizing subscale (Cronbach’s α) was 0.73 in our sample. The externalizing scores (Figure 1A; mean: 5.9, *SD* = 5.3) were positively skewed in our sample with a skewness of 2.39 [standard error (*SE*) = 0.43] and kurtosis of 8.39 (*SE* = 0.85).

#### Callous-Unemotional (CU) Traits

Both caregivers and youth participants filled out their respective versions of the Inventory of Callous Unemotional traits (ICU; Frick, 2004). The ICU is a 24-item questionnaire developed to provide a more comprehensive assessment of CU traits, composited of the callous, uncaring, and unemotional subscales. It is a 4-point rating scale ranging from 0 (not at all true) to 3 (definitely true). The total caregiver-report and self-report CU trait scores were computed separately for each participant. One participant’s self-report data in the callous subscale had one missing item, which was replaced by the average score of the other items in this subscale. Internal consistency of the caregiver-report CU scores for our sample was high (α = 0.89), while the self-report was acceptable (α = 0.61). The greater score of the two reports (e.g., caregiver- vs. self-report) was used to compute the final CU trait score for each participant, as recommended by the ICU and Antisocial Process Screening Device Manual (Frick and Hare, 2001). The CU scores (Figure 1B; mean: 24.9, *SD* = 7.9) were positively skewed in our sample with a skewness of 0.62 (*SE* = 0.43) and kurtosis of −0.59 (*SE* = 0.85).

#### Pubertal Status

Adolescents filled out the Self-Rating Scale for Pubertal Development (Carskadon and Acebo, 1993). Its rating is based on a 4-point scale: 1 (“has not yet begun”), 2 (“has barely”), 3 (“definitely underway”), 4 (“seem complete”), or unknown (i.e., “I don’t know”). It contains three questions for both boys and girls regarding growth in height, body hair growth, and skin changes. Girls answer two additional questions about breast growth and menstruation (and a third question about the age of menstruation, if applicable), while boys answer two additional questions regarding deepening of the voice and facial hair growth. A pubertal status score (prepubertal, early pubertal, mid pubertal, late pubertal, or postpubertal) was computed for each participant based on guidelines from (Crockett, unpublished).

In addition, participants’ IQ scores and social adversity levels were acquired when they were initially recruited. Full-scale IQ was estimated using four subtests of the Wechsler Intelligence Scale for Children-Fourth Edition (WISC-IV; Wechsler, 2003): Verbal Comprehension Index (VCI), Perceptual Reasoning Index (PRI), Working Memory Index (WMI), and Processing Speed Index (PSI). The four-factor indices of the WISC-IV have high reliabilities, ranging from Cronbach’s α of 0.88 to 0.94 (Kaufman et al., 2006). For this study, we assessed VCI using the Vocabulary task, WMI using the Digit Span task, PRI using the Matrix Reasoning task, and PSI using the Coding task. The total scaled score across the three subtests was then converted to the estimated FS-IQ following the Tellegen and Briggs procedure (Tellegen and Briggs, 1967).

A social adversity index was computed from the caregiver’s responses to ten questions based on previous literature (Raine, 2002; Gao et al., 2010a; Zhang and Gao, 2015). A total adversity score was created by adding 1 point for each of the following variables: Divorced Parents (single-parent family, remarriage, or living with guardians other than parents), Foster Home, Public Housing, Welfare Food Stamps, Parent Ever Arrested (either parent has been arrested at least once), Parents Physically Ill, Parents Mentally Ill, Crowded Home (five or more family members per room within the home), Teenage Mother (aged 19 years or younger when the child was born), and Large Family (having five or more siblings by 3 years of age). Items were scored dichotomously with a 0 (no) or 1 (yes). Higher total scores reflect higher social adversity.

### Monetary Incentive Delay (MID) Task

**FIGURE 2:**
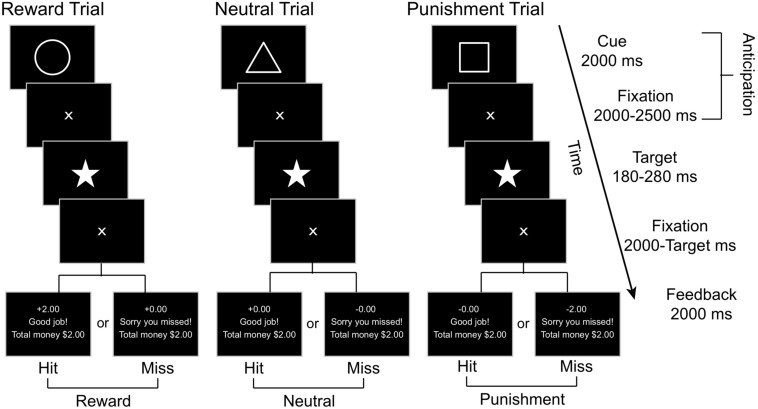
A schematic representation of the MID task, where affectively neutral geometric cues signify the trial type (e.g., reward, neutral, or punishment). Subjects are instructed to press a button as soon as they observe the star target and are given feedback based on their performance toward the end of each trial.

We employed a modified version of the Monetary Incentive Delay Task (Figure 2), adapted from Knutson et al. (2001a) and Samanez-Larkin et al. (2007), to examine the reward and punishment related brain responses. In this task, a Pavlovian conditioning procedure was used with each trial including two phases of interest: anticipation and outcome. The anticipation phase begins with a geometric visual cue displayed for 2000 milliseconds (ms) followed by a 2000–2500 ms central fixation crosshair, with an average total length of 4.26 (*SD* = 0.01, range = 4.0 - 4.5) seconds (s). Each geometric cue was associated with a particular outcome: the circle (reward cue) was associated with potential reward (monetary gain), the square (punishment cue) was associated with potential punishment (monetary loss), and the triangle (neutral cue) was associated with no gain or loss. Participants were explicitly told the meaning of each geometric cue when the task was being explained prior to the practice session. Immediately after the anticipation phase, a pentacle appears on the center of the screen for a short period as a target and participants were required to hit the response button as soon as they detected the target. Only responses made within the window of target duration were considered as correct responses.

For each participant, the initial target duration was set as the mean response time from the 18 practice trials. For each of the following trials, the target duration was adaptively altered based on performance on prior trials to limit the current total hit rates as 66%. A fixation cross was displayed after the target for 2000 ms minus the target duration. Then, in the outcome phase, the feedback was provided for 2000 ms, including response accuracy (“Good job!” for target hit within the time window and “Sorry you missed!” for missed target), together with trial-specific and cumulative rewards earned. If the target was hit within the time window after a reward cue (circle), participants would win $2.00; otherwise, they would gain $0.00. Hit after a punishment cue resulted in losing $0.00, whereas a miss would result in losing $2.00. Hit or miss after a neutral cue resulted in neither gain nor loss (±$0.00). The inter-trial interval was 2000–3000 ms. There were 45 trials in each run, including 15 reward, punishment, and neutral trials each, presented in random order. Each run began with a 15 s fixation period and ended with another 15 s fixation period followed by a feedback of the total rewards earned from the current run. Each run lasted about 9.5 min. There were two runs for each participant, resulting in a total of 90 trials lasting about 19 min.

### fMRI Data Acquisition

MRI acquisitions were obtained on a 3T Siemens Magnetom Skyra scanner with a 32-channel phase-array coil at the ISMMS. Each scan session lasted about 50 min. Foam padding was used to minimize participants’ head movement. All images were acquired along axial planes parallel to the anterior commissure-posterior commissure (AC–PC) line. Two runs of T2^∗^-weighted images for fMRI were acquired during the task with a Multi-band accelerated EPI pulse sequence^1^ with the following parameters: 60 axial slices of 2.4 mm thick, interleaved, skip = 0 mm, TR = 1000 ms, TE = 35 ms, multi-band accel. factor = 6, echo spacing = 0.72 ms, flip angle = 77°, FOV = 228 mm, matrix size = 96 × 96, voxel size = 2.4 mm × 2.4 mm × 2.4 mm. Each run began with a single-band reference image that matched real brain-volumes and acquired without acceleration, followed by 540 volumes covering the task period. A pair of spin-echo echo-planar imaging (SE-EPI) reverse-phase encode field maps were acquired prior to these two runs, with TR = 8600 ms and TE = 65 ms. A high-resolution T1-weighted anatomical volume of the whole brain was acquired after the task with a magnetization-prepared rapid gradient-echo (MPRAGE) sequence with the following parameters: 176 axial slices of 1.0 mm thick, skip = 0 mm, TR = 2400 ms, TE = 1.94 ms, flip angle = 8°, FOV = 256 mm, matrix size = 256 × 256, voxel size = 1.0 mm × 1.0 mm × 1.0 mm.

### Procedure

The task was compiled and executed via E-prime 2.0 software (Psychology Software Tools, Pittsburgh, PA, United States). Stimuli were projected onto a screen placed at the back of the magnet bore and viewed with mirrors mounted on the head coil. Prior to scanning, the task was explained to the adolescents via written and verbal instructions, and then they performed an 18-trial practice session on a PC. A mock scanner with an identical stimulus presentation and response system was shown to each participant to help them acclimate to the MRI environment. MRI-compatible lenses were provided to adolescents who required vision correction. Their responses were collected using a fiber-optic button system with a five-button response glove (BrainLogic, Psychology Software Tools) placed on their dominant hand. Participants were required to make responses by pressing the button under their index finger. At the end of the experiment, participants were debriefed.

### Imaging Preprocessing

Image preprocessing was performed for each participant using the statistical parametric mapping package (SPM 12; Wellcome Trust Center for Neuroimaging, London, United Kingdom; RRID:SCR_007037) and FMRIB Software Library (FSL v6.0^2^; RRID:SCR_002823). The T1 image and all EPI images were manually adjusted to align the AC-PC plane. Bias correction was performed for both T1 and EPI images. Each EPI image volume was then realigned to the first volume and six motion parameters were estimated. Fieldmap in Hz and magnitude images were generated based on the field map images to calculate the voxel displacement map (VDM). The VDM was applied to all EPI images to correct distortions. Head motion and signal drifting were further corrected using the ArtRepair software version 5b (RRID:SCR_005990)^3^. A mean EPI image was calculated across all EPI images after these steps of processing. The brain was extracted from the bias-corrected T1 image and coregistered to the brain extracted from the mean EPI image using normalized mutual information. The coregistered T1 brain was normalized to a bias-corrected 12-years adolescent T1 template (Richards et al., 2016), with affine regularization as ICBM space template – European brains, and resampled to a voxel size of 2 × 2 × 2 mm. Normalized EPI images were then spatially smoothed with a Gaussian kernel of 6 mm full-width half-maximum, as recommended by Sacchet and Knutson (2013) to accurately locate the VST.

### General Linear Modeling (GLM)

The GLM was performed using SPM 12. First-level (single-subject level) statistical analyses of event-related blood oxygenation level-dependent (BOLD) signals were conducted using GLM for each participant. For each run, three regressors were constructed based on the onset vectors of the anticipation phase in three conditions (i.e., Reward, Neutral, and Punishment), with the duration of each event modeled as the total duration of the anticipation phase in the corresponding trial. Six regressors were constructed based on the onset vectors of the outcome phase in six feedback conditions (i.e., Reward-Hit, Reward-Miss, Neutral-Hit, Neutral-Miss, Punishment-Hit, and Punishment-Miss), with the event duration modeled as 0 s. For each of these six regressors, a parametric modulator of the trial-by-trial cumulative rewards earned (demeaned) was constructed to model the influence of this information on brain responses under each feedback condition. Two additional regressors were constructed based on the onset of the targets with hit and missed responses respectively, with the event duration modeled as 0 s. All of these 11 regressors were convolved with a standard hemodynamic response function (HRF). Head motion was modeled as nuisance regressors according to Friston 24-parameter model (Friston et al., 1996), including 6 head motion parameters estimated during realignment, 6 parameters as one time-point before, and 12 corresponding squared items. Low-frequency drifts in signal were removed using a high-pass filter with a 128 s cutoff. Across two runs, one nuisance regressor to indicate runs was entered into the model. The serial correlation was estimated using an autoregressive AR(1) model. This model was estimated for each participant and the images of parameter estimates (β) values were obtained for each regressor. The β images of the three anticipation-associated and six outcome-associated regressors were used in the following analyses.

For the anticipation phase, two contrasts were defined: (1) Reward cue vs. Neutral cue, to examine the involvement in gain-related anticipation, and (2) Punishment cue vs. Neutral cue, to examine the involvement in loss-related anticipation. For the outcome phase, three contrasts were defined: (1) Reward (Hit *minus* Miss) vs. Neutral (Hit *minus* Miss), to examine the involvement in reward receipt (monetary gain), and (2) Punishment (Miss *minus* Hit) vs. Neutral (Miss *minus* Hit), to examine the involvement in punishment receipt (monetary loss). For each of these four effects, a contrast image was generated for each participant.

### Examining the Neural Responses Across the Entire Sample: Whole-Brain Analyses

A second-level group GLM was conducted to examine the neural responses associated with each of these four effects across the entire sample, regardless of the individual differences in externalizing behavior and CU traits. Both positive (increase) and negative (decrease) activation associated with each effect were examined. Age, sex, IQ, social adversity, and pubertal status were entered as covariates in the group-level GLM. In the group-level analysis, we used a cluster-extent thresholding approach to correct for multiple voxel comparisons. Specifically, a threshold consisted of a significance level of *p* < 0.001 (uncorrected) for the height of each voxel (as recommended by Woo et al., 2014), together with a contiguous-voxel extent threshold (*k*; estimated based on the random field theory; Worsley et al., 1992) was adopted, which resulted in a cluster-level *p* < 0.05 threshold.

### Extracting Neural Responses From Regions of Interest (ROI)

We selected two prior defined ROI based on previous meta-analyses for fMRI studies using the MID: ventral striatum (VST), which is associated with reward processing, and amygdala, which is associated with the processing of negative emotion (Knutson and Greer, 2008; Liu et al., 2011; Richards et al., 2013; Silverman et al., 2015; Oldham et al., 2018). These two were defined anatomically. Specifically, the VST was defined according to the Oxford-GSK-Imanova structural striatal atlases (Tziortzi et al., 2011), and the amygdala was defined according to the Harvard-Oxford subcortical structural atlases. We manually traced these two ROIs on the age-specific (12-years-old) anatomical template. Signals in each ROI were defined as the first eigenvariate of the β value from all voxels within the combined cluster of that region in the left and right hemispheres. The ROI signals were extracted from each participant’s first-level contrast map for each of the five effects. The statistical analyses for neural responses in ROIs (see below) are independent of the above group-level GLM analyses across the entire sample. The whole-brain exploratory regression analysis was not conducted because our sample was highly skewed on both externalizing and CU measures and the assumption of normality of variable distribution was not met.

### Modeling the Influence of Externalizing and CU Traits on Neural Responses: Statistical Analyses

#### Primary Measures

The gender difference in both primary measures (externalizing behavior and CU traits) and measures of no interest (age, pubertal status, IQ, and social adversity) were examined using an independent sample *t*-test. Bootstrapped correlations between all measures were also examined. Due to the high skewness of both primary measures, the significance level of each test were estimated using bootstrapping, a non-parametric approach to estimate the population distribution of a statistical value based on the sample distribution. Specifically, a large amount of bootstrapping samples were randomly drawn from the current sample with replacement. The distribution of a statistical value computed based on each bootstrapping sample reflect the population distribution of this value (Wright et al., 2011). The bootstrapping procedure makes fewer assumptions compared to the traditional parametric approaches, and is therefore appropriate for studies with small sample size or with non-normally distributed variables. Here, for each test, 1000 bootstrapping samples were drawn from our sample, and the bias corrected and accelerated 95% confidence intervals (BCa 95% CI) of the statistical values were estimated, which adjusted for bias and skewness in the bootstrapped distribution.

#### Neural Responses

Due to the high skewness of both externalizing and CU scores in our sample, bootstrapping regression (Paparoditis and Politis, 2005) was conducted for the subsequent analyses (number of bootstrapping samples = 1,000, BCa 95% CI was estimated). We examined the direct effect of externalizing behavior and CU traits on ROI activation using a regression model with ROI activation associated with each of the contrast effects defined above as the dependent variable. Age, child sex, IQ, social adversity, and pubertal status were entered as covariates in Step 1, and the primary variable (i.e., externalizing behavior or CU traits) was entered in Step 2 (Model 1). The amount of variance explained by the regressor of interest was estimated as the difference of *R*^2^ between the model and the comparable model in which only all regressors of no interest were included.

If both externalizing and CU scores were significantly associated with ROI activation, we then examined the effect of each primary variable of interest and brain activation when controlling for the non-focal variable by entering it as a regressor of no interest (Model 2). We also compared the *R*^2^ of this model to Model 1 (Δ*R*^2^ = *R*^2^_model 2_ − *R*^2^_model 1_). A reduction of *R*^2^ (negative Δ*R*^2^) indicates a joint contribution of these two variables, while an augment of *R*^2^ (positive Δ*R*^2^) reflects an antagonistic effect.

The joint contribution of externalizing behavior and CU traits on the regional activation was examined using a bootstrapping regression model (Model 3) with covariates of no interest (Step 1), both externalizing behavior and CU traits (Step 2) and their interaction as the predictor of interest (Step 3). The interaction term was computed as the product of these two variables (demeaned). A significant positive coefficient of the interaction term would indicate a superadditive effect of the two (i.e., individuals with higher levels in both CU traits and externalizing behavior are hyper-responsive). A significant negative coefficient of the interaction term would indicate a subadditive effect (i.e., individuals with lower levels of CU traits but higher levels of externalizing behavior are hyper-responsive). Compared to the regression model with only externalizing behavior and CU traits as regressors, an augment of *R*^2^ (positive Δ*R*^2^) of the model with an additional interaction term reflects an incremental contribution of this interaction term on predicting brain activation.

## Results

### Descriptive Statistics of the Measures

The group-mean externalizing and CU scores together with other measures and sex differences are reported in Table 1. Boys and girls did not significantly differ on either externalizing (*p* = 0.430) or CU (*p* = 0.559) scores. Therefore, sex difference was not examined in the subsequent analyses. Boys and girls were also not significantly different on any of the measures of no interest except for pubertal status (*p* = 0.009, with girls showing a higher level of pubertal status than boys).

**TABLE 1:** Group means, standard deviations (SD), ranges, and group-comparisons (by sex) of each measure.

	**All**			**Boys**			**Girls**			**Sex difference**
	***n* = 29**			***n* = 14**			***n* = 15**			
				
	**Mean**	***SD***	**Range**	**Mean**	***SD***	**Range**	**Mean**	***SD***	**Range**	***p***
Age	12.3	0.8	11∼14	12.6	0.6	11∼13	12.0	0.9	11∼14	0.078
IQ	100.7	22.0	59∼154	104.1	24.5	59∼154	97.6	19.7	65∼131	0.431
Social adversity	3.2	2.2	0∼8	2.8	2.2	0∼7	3.7	2.3	0∼8	0.300
Puberty	3.6	0.9	2∼5	3.1	0.6	2∼4	4.0	1.0	3∼5	0.009
Externalizing	5.9	5.3	0∼27	5.0	2.4	0∼9	6.7	7.1	0∼27	0.430
CU	24.9	7.9	13∼40	24.1	7.6	14∼38	25.8	8.3	13∼40	0.556

*CU; callous-unemotional traits.*

Externalizing and CU scores were significantly correlated (*r* = 0.54, BCa 95% CI: 0.264 to 0.778; *p* = 0.039), see Figure 1C, but this correlation became non-significant when controlling all covariates (*r* = 0.58, BCa 95% CI: 0.154 to 0.826; *p* = 0.055). None of the covariates were significantly correlated with externalizing or CU measures except puberty, which was positively correlated with the CU score (*r* = 0.40, BCa 95% CI: 0.086 to 0.694; *p* = 0.031).

### Results of GLM Analysis Regardless of Individual Difference

**FIGURE 3:**
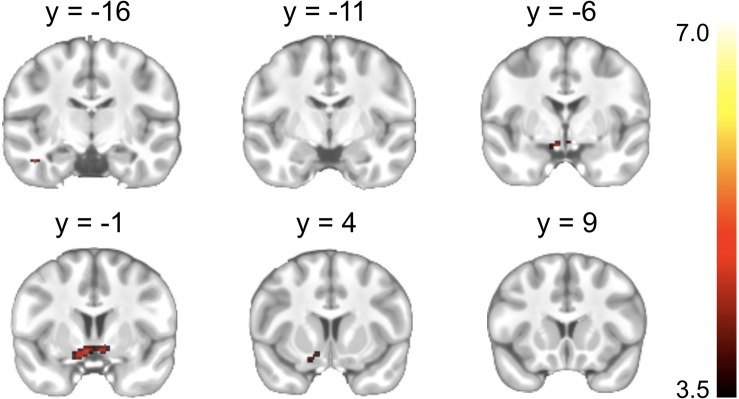
Brain regions showing significant activation changes during the reward anticipation (Reward cue *minus* Neutral cue contrast). Color map indicates the T values.

A significant Reward cue > Neutral cue effect was shown in the VST bilaterally, while no region showed a significant Reward cue < Neutral cue effect (Figure 3). No significant difference between Punishment cue and Neutral cue was found in any brain region. In addition, a significant Reward (Miss *minus* Hit) < Neutral (Miss *minus* Hit) effect was shown in left inferior frontal gyrus, while no region showed a significant Reward (Miss *minus* Hit) > Neutral (Miss *minus* Hit) effect. A significant Punishment (Miss *minus* Hit) < Neutral (Miss *minus* Hit) effect was shown in hippocampus/parahippocampal gyrus bilaterally, while no region showed a significant Punishment (Miss *minus* Hit) > Neutral (Miss *minus* Hit) effect. Coordinates of the peak of each cluster showing significant activation are listed in Table 2.

**TABLE 2:** Brain regions showing significant activation changes in the GLM analysis.

**Regions**	**L/R**	**BA**	***x***	***y***	***z***	***T***	***Z***	***K***
**Reward cue > Neutral cue**								
Ventral striatum	L		−8	−4	−10	4.74	3.92	135
Ventral striatum	R		6	−2	−6	4.46	3.75	
**Feedback: Reward (Hit - Miss) < Neutral (Hit - Miss)**								
Inferior frontal gyrus	L	47	−22	26	−6	5.19	4.18	90
**Feedback: Punishment (Miss - Hit) < Neutral (Miss - Hit)**								
Hippocampus/Parahippocampal gyrus	R	27/35	22	−28	−6	5.66	4.44	226
Hippocampus/Parahippocampal gyrus	L	27/35	−20	−34	−4	5.03	4.09	106

*Regions are listed in a descending order based on their peak *Z* value. For a cluster with multiple local peaks, the number of voxels in the whole cluster (K) was only listed under the first local peak (also for other activation tables). The threshold was *p* < 0.001 for the height and cluster level *p* < 0.05. L, left; R, right. BA, Brodmann area.*

### Results of ROI Analysis

#### Reward-Related Anticipation

**FIGURE 4:**
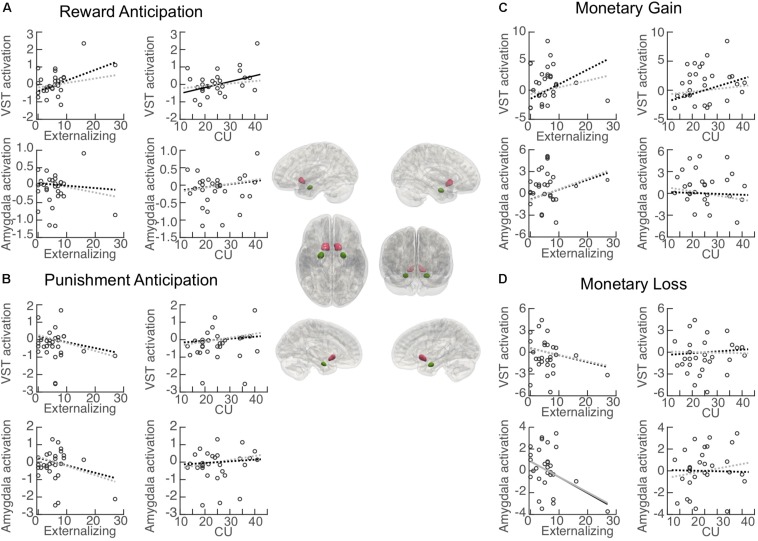
Association between primary measures and activation in regions of interest (ROI). Central panel: Illustration of the anatomically defined ROI of the ventral striatum (VST) and amygdala. Correlation (black lines) and partial correlation (gray lines) between externalizing behavior/CU traits and ROI activation during **(A)** reward anticipation (Reward cue minus Neutral cue); **(B)** punishment anticipation (Punishment cue minus Neutral cue); **(C)** reward (monetary gain) [Reward (Hit *minus* Miss) minus Neutral (Hit *minus* Miss)], and **(D)** punishment (monetary loss) [Punishment (Miss *minus* Hit) minus Neutral (Miss *minus* Hit)]. Solid lines: significant association. Dashed lines: non-significant association.

Associations between each primary variable of interest (i.e., externalizing behavior and CU traits) and activation in each ROI during reward anticipation are illustrated in Figure 4A. Activation in the VST was significantly positively associated with CU traits (β = 0.053, BCa 95% CI: 0.008 to 0.095; *p* = 0.046), but no significant association was found for externalizing behavior (β = 0.065, BCa 95% CI: −0.023 to 0.159; *p* = 0.071). When controlling for externalizing score, the CU-VST activation relationship became non-significant (β = 0.042, BCa 95% CI: −0.018 to 0.111; *p* = 0.166, Δ*R*^2^ = −0.17). Similarly, when controlling for CU, the externalizing–VST relationship was non-significant (β = 0.033, BCa 95% CI: −0.005 to 0.074; *p* = 0.188, Δ*R*^2^ = −0.17). These findings suggest that there was a substantial overlap between the contributions of CU and externalizing to VST activation. Externalizing behavior and CU traits explained 23.3 and 22.8% of the variance of the activation in the VST, respectively. The externalizing by CU interaction term in the moderation model was not significant (β = 0.08, BCa 95% CI: −0.008 to 0.020; *p* = 0.186).

Activation in the amygdala was not significantly associated with either externalizing behavior (β = −0.007, BCa 95% CI: −0.041 to 0.068; *p* = 0.822) or CU traits (β = 0.014, BCa 95% CI: −0.017 to 0.045; *p* = 0.366). The externalizing behavior by CU interaction term in the moderation model was not significant (β = 0.004, BCa 95% CI: −0.006 to 0.020; *p* = 0.358).

#### Punishment-Related Anticipation

Associations between externalizing behavior/CU traits and activation in each ROI during punishment anticipation are illustrated in Figure 4B. Activation in the VST was not associated with either externalizing behavior (β = −0.036, BCa 95% CI: −0.103 to 0.076; *p* = 0.118) or CU traits (β = 0.018, BCa 95% CI: −0.035 to 0.084; *p* = 0.523). Externalizing behavior and CU traits explained 4.6 and 1.7% of the variance of the activation in the VST, respectively. The externalizing behavior by CU interaction effect in the moderation model was not significant (β = −0.001, BCa 95% CI: −0.016 to 0.022; *p* = 0.858).

Activation in the amygdala was not significantly associated with either externalizing behavior (β = −0.045, BCa 95% CI: −0.090 to 0.060; *p* = 0.262) or CU traits (β = 0.015, BCa 95% CI: −0.045 to 0.074; *p* = 0.616). Externalizing behavior and CU traits explained 6.9 and 1.1% of the variance of the activation in the amygdala, respectively. The externalizing behavior by CU interaction effect in the moderation model was not significant (β = −0.003, BCa 95% CI: −0.017 to 0.033; *p* = 0.677).

#### Reward Receipt (Monetary Gain) Related Responses

Associations between externalizing behavior/CU and activation in each ROI during the receipt of reward (monetary gain) are illustrated in Figure 4C. Activation in the VST was not significantly associated with either externalizing behavior (β = 0.266, BCa 95% CI: −0.173 to 0.502; *p* = 0.058) or CU traits (β = 0.202, BCa 95% CI: −0.066 to 0.507; *p* = 0.119). Externalizing behavior and CU traits explained 17.0 and 14.4% of the variance of the activation in the VST, respectively. The externalizing behavior by CU interaction effect in the moderation model was not significant (β = −0.006, BCa 95% CI: −0.053 to 0.030; *p* = 0.785).

Activation in the amygdala was not significantly associated with either externalizing behavior (β = 0.142, BCa 95% CI: −0.143 to 0.241; *p* = 0.137) or CU traits (β = −0.019, BCa 95% CI: −0.183 to 0.144; *p* = 0.808). Externalizing behavior and CU traits explained 8.6 and 0.2% of the variance of the activation in the amygdala, respectively. The externalizing behavior by CU interaction effect in the moderation model was not significant (β = 0.004, BCa 95% CI: −0.046 to 0.033; *p* = 0.820).

#### Punishment Receipt (Monetary Loss) Related Responses

Associations between externalizing behavior/CU and activation in each ROI during the receipt of punishment (monetary loss) are illustrated in Figure 4D. Activation in the VST was not significantly associated with either externalizing behavior (β = −0.101, BCa 95% CI: −0.289 to 0.086; *p* = 0.275) or CU traits (β = −0.011, BCa 95% CI: −0.170 to 0.147; *p* = 0.883). Externalizing behavior and CU traits explained 4.9 and 0.1% of the variance of the activation in the VST, respectively. The externalizing behavior by CU interaction effect in the moderation model was not significant (β = 0.025, BCa 95% CI: −0.030 to 0.089; *p* = 0.095).

Activation in the amygdala was significantly associated with externalizing behavior (β = −0.155, BCa 95% CI: −0.305 to −0.006; *p* = 0.042), and remained significant after controlling for CU (β = −0.226, BCa 95% CI: −0.375 to −0.091; *p* = 0.004, Δ*R*^2^ = 0.06). CU traits were not significantly associated with activation in the amygdala (β = −0.008, BCa 95% CI: −0.167 to 184; *p* = 0.903). Externalizing behavior and CU traits explained 14.6 and 0.1% of the variance of the activation in the amygdala, respectively. The externalizing behavior by CU interaction effect in the moderation model was not significant (β = 0.007, BCa 95% CI: −0.021 to 0.044; *p* = 0.583).

## Discussion

The present study examined the neural mechanisms of reward and punishment processing in relation to externalizing behavior and CU traits (individually and jointly) in a community sample of adolescents. Findings provide partial support for the theories of hyper-responsivity to reward and hypo-responsivity to punishment in CU and externalizing youths.

Partially consistent with our hypothesis of hyper-responsivity to reward in youths with high externalizing behavior and/or CU traits, CU traits were positively correlated with VST activation during reward anticipation. Similar positive relationship with VST activation was also found for externalizing behavior, although it was non-significant (*p* = 0.071), likely due to the small sample size. We also demonstrated that externalizing behavior and CU traits share substantial overlap in predicting activation of the VST during reward anticipation, given that once the effect of the non-focal variable was taken into account, the associations became non-significant. Abnormal anticipatory responses in the VST have been suggested as a biomarker for various high-risk (e.g., impulsive, addicted) populations (see Balodis and Potenza, 2015 for a review), particularly in the context of reward anticipation. In contrast, some psychiatric (e.g., schizophrenia, depression) conditions have been associated with blunted activation in the VST (Knutson and Heinz, 2015). Taken together, hyperactivity in the VST may be potentially used to predict future problematic and risky behaviors that are reward dominant, including externalizing behavior, CU traits, substance abuse, and gambling (Blaszczynski et al., 1997; King et al., 2004).

Partly in line with our hypothesis of hypo-responsivity to punishment, we found that externalizing scores were negatively associated with the amygdala responses to monetary loss during punishment receipt (with or without controlling CU traits). Specifically, adolescents with fewer externalizing behaviors showed stronger amygdala activation when the outcome of missing the target was accompanied by a punishment as monetary loss (Miss *minus* Hit after the punishment cue), compared to the same outcome but with no punishment (Miss *minus* Hit after the neutral cue). However, for individuals with higher externalizing scores, they showed weaker amygdala activation when missing the target was accompanied by a punishment (see Figure 4B), suggesting that the neural processing of punishment may be suppressed in adolescents with more externalizing behaviors. These results are similar to previous research study on DBD youth (e.g., Finger et al., 2011) that found amygdala deactivation in response to punishment receipt.

In addition, no significant interaction effect between CU and externalizing was found for any of the ROIs when anticipating or receiving rewards or punishment, failing to support our hypothesis that individuals with higher levels of both externalizing behavior and CU traits would show the most salient abnormalities in brain activation. It is worth noting that our study is the first to examine the interaction effects of CU and externalizing behavior on reward/punishment anticipation and processing. Prior functional brain imaging studies that examined additive effects of CU and externalizing measures (e.g., Herpertz et al., 2008; Passamonti et al., 2010) had focused on neural processing of emotional stimuli. In addition, interactive effects of CU and externalizing measures seem to be more prominent in the amygdala activation during fear-related processing in particular (Viding et al., 2012; Cardinale et al., 2018). Finally, our null findings may also be due to low statistical power, and future studies with larger sample size should be conducted to detect if any interaction effects may exist using reward and punishment-related paradigms.

Alternatively, the lack of effect may be partly due to the nature of the task (e.g., more anticipation events relative to feedback), which ultimately makes it less powerful for detecting effects for reward receipt. Relatedly, the MID task has been extensively used to assess reward processing, but less for punishment processing (Oldham et al., 2018). Potentially, this task may be less sensitive to punishment anticipation than to reward anticipation. In fact, studies utilizing the MID have more often linked personality traits to VST activation to reward than to punishment (e.g., Buckholtz et al., 2010; Bjork et al., 2012).

The current research also has caveats to consider. First, the sample size is small, limiting statistical power. Moreover, the externalizing and CU scores were highly skewed in our sample. Two participants had very high scores on these measures, with one having an externalizing score (27) greater than 2 SD (but less than 3 SD) of the group mean, and the other having a CU score (41) greater than 2 SD (but less than 3 SD) of the group mean. Normative samples of adolescents aged 12–14 years have reported CBCL externalizing behavior means of 7.01 and 5.38 for boys and girls, respectively (Bongers et al., 2003). Means for CU traits (via the ICU) range from 23.45 to 31.05 in community adolescent samples (Roose et al., 2010; Feilhauer et al., 2012). Still, we caution that our results may be less generalizable to individuals with clinical diagnosis of DBD because our range of externalizing scores (expect the one with very high score) is rather limited (e.g., 0–16), whereas prior reports of externalizing behavior for adolescent in clinical samples range from 18.92 to 27.2 (Bögels et al., 2008; Bjork et al., 2010; Schaeffer et al., 2014). Similarly, ranges for CU traits in our sample (after excluding the outliers) were diverse (13–38, mean = 23.8), while detainee and offender samples report total score means ranging from 23 to 41 (Kimonis et al., 2008, 2013; White et al., 2013). Next, although we did incorporate both sexes in our study, boys and girls did not significantly differ on externalizing behavior or CU traits in our sample. This may be due to low statistical power resulted from a small sample size. Sex differences have been found in the gray matter volumes of the OFC (Raine et al., 2011) and volumetric brain asymmetries of the OFC and ACC (Visser et al., 2013) in antisocial populations. Future work with larger sex-mixed samples is needed to determine the effects of sex, especially since there are large gender gaps for prevalence rates of externalizing disorders (Newman et al., 1996; Hicks et al., 2007). Finally, we only focused on the VST and amygdala as our ROIs because of their well-documented involvement in the reward and punishment processing (Bjork et al., 2012; Cohn et al., 2015; Oldham et al., 2018). Although the involvement of other regions including the vmPFC and insula has been reported (Knutson et al., 2001b; Balodis et al., 2012; Silverman et al., 2015; Oldham et al., 2018), our exploratory whole-brain regression analysis did not reveal any significant effects for these areas in our sample.

One strength of our work is that we recruited from an ethnically diverse and mixed-sex healthy community sample. Previous studies have primarily been on male and clinical populations (e.g., Finger et al., 2011; Bjork et al., 2012; Pujara et al., 2013; Cohn et al., 2015; Byrd et al., 2018). Only recently has the focus shifted to the inclusion of community samples with both sexes and younger age groups, which is what we were able to accomplish in this study. Taken together, our results suggest that both externalizing behavior and CU traits are associated with hyper-responsivity to reward, and that externalizing behavior in particular is associated with hypo-responsivity to punishment. As both externalizing behavior and CU traits in youth are known risk factors for criminal offending in adulthood (Hinshaw and Lee, 2003; Frick and White, 2008), it may be beneficial for future work to evaluate the degree to which externalizing problems and CU traits are independently and jointly associated with neural activity. This knowledge will help us better understand the etiological basis of externalizing problems and CU traits and eventually contribute to interventions for these unwanted trajectories.

## Data Availability Statement

The datasets generated for this study are available on request to the corresponding author.

## Ethics Statement

The studies involving human participants were reviewed and approved by the Institutional Review Board of Brooklyn College (CUNY). Written informed consent to participate in this study was provided by the participants’ legal guardian/next of kin.

## Author Contributions

YH and YG conceived and planned the research study. YH, SF, and SL carried out the study. YH and TW preprocessed the data. YL, ZW, and XL contributed to the imaging preprocessing pipeline. TW performed the statistical analyses. YH, TW, and YG wrote the manuscript with input from all other authors. All authors provided critical feedback to all components of the research.

## Conflict of Interest

The authors declare that the research was conducted in the absence of any commercial or financial relationships that could be construed as a potential conflict of interest.

## Footnotes

1 https://www.cmrr.umn.edu/multiband/

2 https://fsl.fmrib.ox.ac.uk/fsl/fslwiki/

3 https://cibsr.stanford.edu/tools/human-brain-project/artrepair-software.html
